# Association Between CD147 Expression, *RAS* Mutational Status, and Local Recurrence in Resected Locally Advanced Rectal Cancer

**DOI:** 10.1002/cam4.71087

**Published:** 2025-07-29

**Authors:** Anaëlle Isnard, Laure‐Amandine Cramier, Rémi Vergara, Mehdi Boubaddi, Donatien Fouche, Benjamin Fernandez, Nathalie Senant, Quentin Denost, Éric Rullier, Pierre Dubus, Sandrine Dabernat, Charles Dupin, Véronique Vendrely, Anne Rullier, Samuel Amintas

**Affiliations:** ^1^ CHU Bordeaux, Tumor Biology and Tumor Bank Laboratory Pessac France; ^2^ BRIC (BoRdeaux Institute of onCology), UMR1312, INSERM, University of Bordeaux Bordeaux France; ^3^ Surgical Pathology Department CHU Bordeaux Bordeaux France; ^4^ Digestive Surgery Department CHU Bordeaux Pessac France; ^5^ Histopathology Platform, TBM‐Core, University of Bordeaux Bordeaux France; ^6^ Bordeaux Colorectal Institute Bordeaux France; ^7^ Biochemistry Laboratory, CHU Bordeaux Bordeaux France; ^8^ Radiotherapy Department CHU Bordeaux Pessac France

**Keywords:** BRAF, CD147, EMMPRIN, gender, KRAS, local recurrence, rectal cancer

## Abstract

**Aim:**

About 5% to 10% of rectal cancer (RC) patients experience local disease recurrence after chemoradiotherapy (CRT) and surgery. Identifying patients at high risk of local recurrence (LR) could lead to the personalized management of the patients. Multiple clinicopathological parameters are associated with LR. However, the potential of tumors for local regrowth may partially lie in the expression of pro‐tumoral factors and genetic characteristics. In this work, we aimed to evaluate the association of tumor CD147 expression, a well‐described cancer aggressiveness biomarker, and mutational status, with post‐resection RC LR.

**Methods:**

We retrospectively analyzed all patients experiencing LR after CRT and resection at Bordeaux University Hospital (2010 to 2019) and matched a LR‐negative control cohort. CD147 expression was evaluated by immunohistochemistry on post‐neoadjuvant treatment residual tumors. Mutational status was assessed by next‐generation sequencing. Correlations with LR rates were evaluated.

**Results:**

The analysis included 29 patients with recurrences and 60 without. The pathological node invasion stage (*p* = 0.023) and the median number of invaded nodes (*p* = 0.008) were significantly associated with LR. CD147 expression and mutational status were not associated with LR. Nonetheless, a notable association was observed between *RAS* mutation and elevated CD147 expression, particularly in male patients (*p* = 0.005). We confirmed this association in an independent validation cohort of 86 RC patients.

**Conclusion:**

CD147 tumor expression levels and mutational status do not appear to be associated with resected RC LR. However, the association between *RAS* mutational status and CD147 expression in male patients tends to confirm the relationship between *RAS* activating mutations and tumor aggressiveness, as well as their sex‐related impact on colorectal tumor biology.

## 1 Introduction

With specific localization, rectal cancer (RC) represents one‐third of all colorectal cancers (CRC) [1]. Historically, locally advanced RC has been characterized by high local and distant recurrence rates [2]. During the last 20 years, the standardization of RC multimodal management allowed significant progress. In particular, the introduction of total neoadjuvant treatment, including chemoradiotherapy (nCRT) and neoadjuvant chemotherapy, combined with surgical resection, including total mesorectal excision (TME), reduced the average local and distant recurrence rates to 4.8% and 21.2%, respectively [3]. Besides clinical staging and evaluation of circumferential margin by magnetic resonance imaging (MRI), biological markers could help identify patients who are at the highest risk of recurrence and provide them with more aggressive therapy, while sparing others from unnecessary treatments.

Pathological complete response (pCR) appears as the major predictor of recurrence. However, only 20% to 25% of RCs show pCR, and few recurrence‐predictive markers are currently available for tumors exhibiting incomplete response to nCRT, especially for local recurrences (LR), which are less frequent. Incomplete microscopic (R1) or macroscopic (R2) resection related to positivity of the circumferential resection margin represents an important risk factor for LR and is associated with a poorer prognosis [4]. Interestingly, the risk of incomplete resection can be accurately predicted by the circumferential margin assessed by MRI [5]. Other pathological tumor‐related characteristics, including advanced pathological tumor stage (pT stage), presence of invaded lymph nodes (pN stage), lymphovascular or perineural invasion, and poor differentiation, are predictive for the development of LR [4, 6]. Seric tumor markers, such as CEA and CA19‐9, although not specific, have shown a correlation with the risk of recurrence, particularly when serum levels are still elevated postoperatively [7].

However, the likelihood of rectal tumors' LR might depend, at least partially, on the expression of pro‐invasive factors or the presence of distinct somatic genetic characteristics. Matrix metalloproteinases (MMPs) are widely involved in the invasion and metastasis process of malignant tumors, in particular by reshaping the extracellular matrix [8]. CD147 factor, also known as EMMPRIN or Basigin, represents a key factor for MMPs recruitment and activation. CD147 protein expression increases in tumors relative to associated healthy tissues and is associated with poorer prognosis in several tumor types, including CRC [9]. In addition, high CD147 protein expression in hepatocellular tumors has been linked with recurrence after liver transplantation [10]. CD147 expression could therefore represent a potential predictive factor of RC recurrence.

On the other hand, the molecular mutational status of main CRC oncogenes (*KRAS, NRAS, BRAF, PIK3CA*), associated with microsatellite instability (MSI) status, is now performed in clinical routine for the management of CRC. The presence of oncogene‐activating mutations impacts CRC tumor biology as well as patients' pathological and clinical parameters [11]. *KRAS* mutations are linked to metastatic CRC's advanced disease stage, poor tumor differentiation, distant metastasis, and poorer prognosis [12]. In nonmetastatic patients, the *KRAS* mutation leads to a more aggressive disease, with faster relapse, and is associated with pulmonary recurrences [13]. Additionally, the presence of an associated *PIK3CA* mutation worsens the prognosis [14]. *BRAF* mutations are also associated with a poorer prognosis, particularly in the metastatic setting [15]. Thus, these mutations could be confounding factors and should not be ignored when evaluating the role of CD147 in rectal tumor LR.

In this work, we investigated if the expression of CD147, as well as the mutational status of residual tumor rectal samples, could be associated with the rectal tumor recurrence after neoadjuvant chemoradiotherapy and curative surgery and thus could be used as a biomarker for LR risk stratification.

## 2 Methods

### 2.1 Patients' Inclusion and Follow‐Up

The flowchart for patient inclusion is shown in Figure S1. Briefly, patients with locally advanced RC treated and operated on between January 2010 and December 2019 at Bordeaux University Hospital, and presented LR, with or without associated metastatic recurrence, were included. LR was defined as the development of any lesion within the operative field after the initial resection and with biopsy‐proven rectal adenocarcinoma recurrence. All patients received a neoadjuvant treatment. As our work focuses on residual tumor specimens, patients presenting a pCR on resection specimens were excluded (*n* = 1), as well as mucinous/colloïd response (> 90%, *n* = 2) because of the low rate of residual cancer cells. We matched a control patient's cohort with a 1:2 ratio based on age (dichotomized at 65 years), sex, MRI, and rectoscopy‐assessed cTN stage before nCRT, R0/R1 resection status, and presence of adjuvant treatment. We have taken into account the date of resection to obtain comparable median follow‐up and age of the samples between the two groups. Patients who died or were lost to follow‐up before 48 months of follow‐up were excluded from the control cohort. The minimum follow‐up time was 3 years. Patients with a lack of sufficient residual tumor tissue were excluded. We calculated, for all patients, an adapted PREDICT Score, substituting the < or ≥ 2 mm circumferential resection margin status by R0/R1 status (Table S1). All patients signed informed consent. This work was performed following the human and ethical principles of research outlined in the Helsinki guidelines and following local statutory requirements (Acceptance of the study by the Bordeaux University Hospital ethics review board on 28/12/2024, reference CER‐BDX 2023‐128).

### 2.2 Immunohistochemistry Procedure

A formalin‐fixed, paraffin‐embedded (FFPE) sample of each residual tumor from the surgical rectal specimen was selected per patient. A 4‐μm‐thick section from each block was deparaffinized and gradually rehydrated before antigen heat retrieval (sodium citrate 10 mM solution for 20 min at 98°C in PT‐Link; Agilent). Immunohistochemistry was performed using the DakoCytomation Autostainer Plus (Agilent, Santa Clare, USA). Inhibition of endogenous peroxidase activity was carried out with the Agilent kit Flex Peroxidase Block solution for 10 min at room temperature. The sections were then incubated with a primary monoclonal anti‐CD147 (8D6; Santa Cruz Biotechnology, USA) for 45 min at room temperature. The sections were incubated with EnVision FLEX+ Mouse (LINKER) for 20 min at room temperature. The signal was amplified using an Envison‐FLEX detection system for 20 min at room temperature. Staining was visualized using a chromogenic solution of 3,3′‐diaminobenzidine for 10 min at room temperature. Counterstaining with hematoxylin solution was performed using the Leica ST5020 CV5030 automated stainer (Leica Biosystems, Wetzlar, Germany). For the negative controls, primary antibodies were replaced with PBS. We used a unique CRC tumor sample as a positive control for each staining run.

### 2.3 Immunohistochemistry Analysis

CD147 immunostaining was analyzed microscopically by two independent, trained pathologists who were blind to the clinical data. The evaluation of CD147 immunostaining was assessed, considering both the percentage of positive tumor cells and the staining intensity on the whole slide, adapted from breast cancer [16]. The percentage of positive tumor cells was scored semiquantitatively as 0 (negative), 1 (< 25%), 2 (25%–49%), and 3 (≥ 50%). Staining intensity was classified as 0 (colorless), 1 (pallid), 2 (yellow), and 3 (brown). The score was calculated as the sum of the percentage of positive tumor cells and staining intensity scores, leading to a maximal score value of 6. The expression was set to “null” (0), “low” (1, 2), “medium” (3, 4), and “high” (5, 6) (Table S2). For each cohort, the mean score was calculated and rounded to the nearest whole number, and a binary classification was then applied: Scores below the mean were categorized as negative; while scores equal to or above the mean were classified as positive CD147 expression.

### 2.4 Next‐Generation Sequencing

Tumor DNA was extracted from a 1‐mm punch made in the FFPE tumor block previously selected for CD147 immunostaining using a Maxwell RSC DNA FFPE Kit (Promega, Charbonnières‐les‐Bains, France). Targeted next‐generation sequencing was performed as previously described [17]. Mutations identified in tumors for *KRAS*, *NRAS*, *BRAF*, and *PIK3CA* genes are summarized in Table S3.

### 2.5 Validation Cohort

The independent validation cohort was established with 86 locally advanced RC patients who underwent tumor surgical resection between January 2012 and December 2024 at Bordeaux University Hospital. The *RAS/BRAF* mutational status of these tumors was determined as part of routine clinical care using NGS or targeted assays, and CD147 immunohistochemical analysis was performed on resection specimens. No additional specific inclusion or exclusion criteria were applied. An independent pathologist conducted the microscopic analyses in a blinded manner, with the same scoring evaluation as the initial cohort. Table S4 provides an overview of the cohort's key clinicopathological characteristics, along with CD147 IHC expression and *RAS/BRAF* mutation status.

### 2.6 Statistical Analysis

For quantitative parameters, a nonparametric Mann–Whitney *U* test was used to compare continuous variables expressed as means and standard deviations. Comparisons of categorical variables among nonrecurrent and local recurrent cancer groups were performed using the χ^2^ (Pearson's chi‐square) tests and Fisher's exact tests. A *p* < 0.05 was considered statistically significant. All statistical analyses were performed using R Studio software.

## 3 Results

### 3.1 Patient Demographics

In total, 89 patients were included, with 29 locally recurrent RC patients and 60 nonrecurrent patients. Demographic and clinicopathological parameters of the two groups are summarized in Table 1. Briefly, patients' cohorts combined 61 (68%) males and 28 females (32%), and the mean age was 64.7 ± 12.3 years. Most of the patients (72%, 68/89) received conventional radiochemotherapy protocol (50Gy + capecitabine) and were operated on with TME procedure (78%, 69/89). Twenty‐four patients had a locoregional pelvic recurrence, and the remaining five had localized intraluminal recurrence (Table S5). Sixteen patients had associated metastatic disease, single or multiple, synchronous or metachronous, with LR (Table S6). Regarding the association of LR and clinicopathological patient features, the pathological nodal metastasis status (ypN) was significantly associated with the LR (*p* = 0.029) (Table 1). This difference is driven by the higher proportion of ypN2 in the recurrent RC group (*p* = 0.006). Following this result, the median number of positive lymph nodes was significantly higher for patients with LR (2.4 ± 3.5 nodes involved) than nonrecurrent ones (0.9 ± 1.5 nodes) (Table S7).

**TABLE 1:** Patients' demographic and clinicopathological features and their association with local recurrence.

All patients (*n* = 89)			
Clinicopathological features	Recurrent (*n* = 29)	Nonrecurrent (*n* = 60)	*p*
	No of patients (%)	No of patients (%)	
**Age, years** ^a^			
< 65	16 (55)	21 (35)	0.07
≥ 65	13 (45)	39 (65)	
**Sex** ^a^			
Male	21 (72)	40 (67)	0.584
Female	8 (28)	20 (33)	
**Distant tumor from anal verge (cm)**			
< 5	15 (52)	30 (50)	0.879
≥ 5	14 (48)	30 (50)	
**Clinical tumor size (cm)**			
< 5	16 (55)	24 (44)	0.314
≥ 5	13 (45)	31 (56)	
Unknown	0	5	
**cT stage** ^a^			
cT2	4 (14)	10 (17)	0.335
cT3	16 (55)	40 (66)	
cT4	9 (31)	10 (17)	
**cN stage** ^a^			
cN0	4 (14)	6 (10)	0.869
cN1	21 (72)	45 (75)	
cN2	4 (14)	9 (15)	
**ypT stage**			
ypT1	2 (7)	5 (8)	0.626
ypT2	6 (21)	20 (33)	
ypT3	18 (62)	31 (52)	
ypT4	3 (10)	4 (7)	
**ypN stage**			
ypN0	13 (52)	31 (54)	0.023*
ypN1	5 (20)	22 (39)	
ypN2	7 (28)	4 (7)	
ypNx (Local excision)	4	3	
**nCRT**			
RTCT	18 (62)	50 (83)	0.299
CT + RTCT	6 (21)	6 (10)	
Others	5 (17)	4 (7)	
**Surgery type**			
TME	21 (72)	48 (80)	0.274
LE	4 (14)	2 (3)	
APR	2 (7)	7 (12)	
PE	2 (7)	3 (5)	
**Anatomopathological tumor size (cm)**			
< 3	13 (45)	33 (55)	0.368
≥ 3	16 (55)	27 (45)	
**Differenciation**			
Well‐moderate	24 (83)	56 (93)	0.121
Poor	5 (17)	4 (7)	
**Resection** ^a^			
R0	17 (59)	38 (63)	0.668
R1	12 (41)	22 (37)	
**Vascular or nervous invasion**			
No	16 (55)	41 (68)	0.225
Yes	13 (45)	19 (32)	
Vascular invasion	4 (14)	9 (16)	0.54
Nervous invasion	3 (10)	5 (8)	
Both	6 (21)	5 (8)	
**Distal resection margins status**			
Negative	22 (88)	55 (95)	0.359
Positive	3 (12)	3 (5)	
NA (local excision)	4	3	
**Postoperative treatment** ^a^			
No	14 (48)	36 (60)	0.296
Yes	15 (52)	24 (40)	
Capecitabine	1 (7)	7 (29)	0.09
FOLFOX	14 (93)	17 (71)	
**Adapted PREDICT score**			
Low	9 (36)	27 (47)	0.203
Moderate	11 (44)	26 (46)	
High	5 (20)	4 (7)	

Abbreviations: APR, abdominoperineal resection; CT + RTCT, induction chemotherapy before RTCT; LE, local excision; nCRT, neoadjuvant radiochemotherapy; PE, pelvic exenteration; RTCT, radiochemotherapy 50Gy + capecitabine; TME, total mesorectum excision.

^a^ Clinicopathological parameters used for control cohort matching.*Statistically significant *p*‐values.

### 3.2 CD147 Expression in Residual Rectal Adenocarcinoma

**FIGURE 1:**
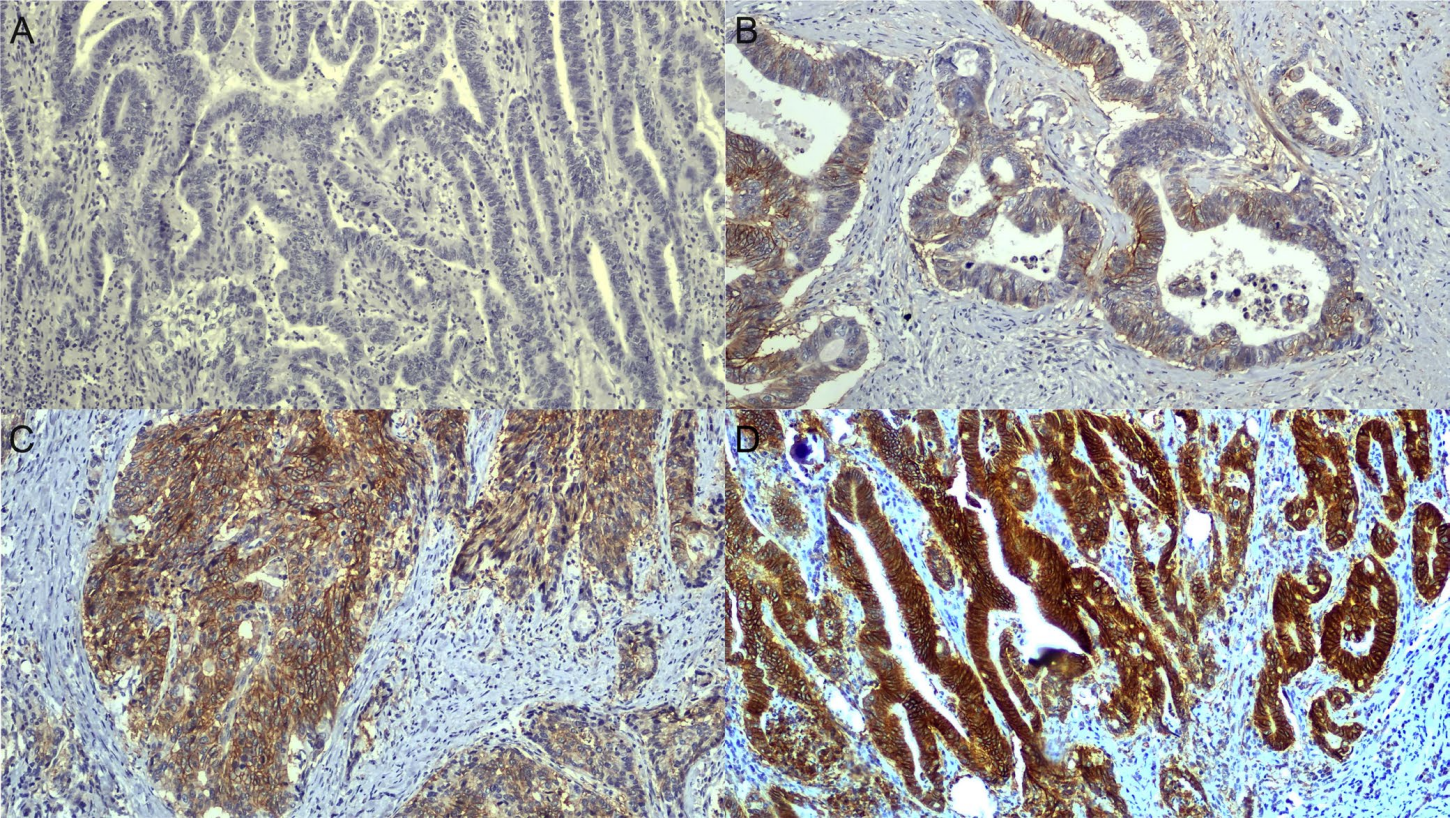
Immunohistochemical staining of CD147 in rectal cancer tissues. Panels (A–D) illustrate the four levels of staining intensity observed for CD147 using the clone 8D6 antibody. (A) No detectable staining (colorless/null), (B) low staining intensity with weak and focal staining, (C) moderate and diffuse staining intensity, and (D) high/strong and widespread staining intensity. Staining was performed on formalin‐fixed, paraffin‐embedded tissue sections, with hematoxylin counterstaining. Magnification: ×100.

Immunohistochemical CD147 staining showed membranous and cytoplasmic staining of tumor cells (Figure 1A–D). Staining was considered positive (medium/high expression) for 13 of 29 (45%) patients of the LR‐positive group and 39 of 60 (65%) patients of the LR‐negative group (Table 2). No significant difference was established for CD147 tumor expression distribution between the two groups.

**TABLE 2:** Association of recurrence and CD147 immunohistochemical expression of rectal tumor.

All patients (*n* = 89)			
CD147 IHC expression	Recurrent (*n* = 29)	Nonrecurrent (*n* = 60)	*p*
	No of patients (%)	No of patients (%)	
Positive	13 (45)	39 (65)	0.07
Negative	16 (55)	21 (35)	

Abbreviation: IHC, immunohistochemistry.

### 3.3 Mutational Status in Rectal Adenocarcinoma and Association With Clinicopathological Parameters

NGS analysis was valid for 83 cases (93%). Table S8 recapitulates cohort mutation distribution according to sex. Women presented a significantly higher rate of *RAS* (*KRAS or NRAS*) mutations (*p* = 0.018). No significant differences in the distribution were observed for the presence of an oncogene‐activating mutation (*p* = 0.62), a *RAS* mutation (*p* = 0.095), and a *PIK3CA* mutation (*p* = 0.381), nor for the number of mutations (*p* = 0.265), between LR‐positive and negative groups (Table S9).

### 3.4 CD147 Expression and Mutational Status in Rectal Adenocarcinoma

Given that CD147 expression, as well as mutational status, impacts colorectal tumor prognosis, we further investigate the association of these two parameters. No significant correlation was observed between CD147 expression and the presence of a *PIK3CA* mutation (*p* = 0.301), or the number of oncogenes mutations (*p* = 0.066; Table 3). However, we highlighted a significant association between the expression of CD147 and the presence of an oncogenic mutation (*p* = 0.035). This association remained consistent concerning *RAS* mutations only (*p* = 0.049; Table 3). Interestingly, it was strengthened in the male patients' group (*p* = 0.005; Table 4), and absent in the female (*p* = 0.653; Table S10). To further explore this observation, we performed CD147 immunohistochemical analysis on 86 additional RC surgical specimens, for which the mutational status of *KRAS, NRAS*, and *BRAF* genes had already been determined as part of routine clinical practice. The results from this validation cohort further tend to support our initial observation. Indeed, no significant association was found between *RAS* mutational status and CD147 expression in female tumors (*p* = 0.519; Table S11). However, in male patients, a significant association emerged (*p* = 0.048; Table S12), in accordance with the previous result. Interestingly, the association became more pronounced when oncogenic *BRAF* mutations were taken into account (*p* = 0.012; Table S13). Taken together, our results suggest that male *RAS*‐mutated rectal tumors express CD147 to a greater extent.

**TABLE 3:** Association of CD147 immunohistochemical expression and mutation status of rectal tumor.

All patients (*n* = 89)			
Molecular features	CD147 IHC negative (*n* = 37)	CD147 IHC positive (*n* = 52)	*p*
	No of patients (%)	No of patients (%)	
**Mutation**			
No	19 (53)	16 (33)	0.035*
Yes	15 (44)	33 (67)	
NI	3	3	
**Mutation number**			
0	19 (56)	16 (33)	0.066
1	13 (38)	24 (49)	
≥ 2	2 (6)	9 (18)	
NI	3	3	
**RAS Mutation**			
No	22 (65)	21 (43)	0.049*
Yes	12 (35)	28 (57)	
NI	3	3	
**PIK3CA mutation**			
No	30 (88)	39 (80)	0.301
Yes	4 (12)	10 (20)	
NI	3	3	

Abbreviation: IHC, immunohistochemistry.

* Statistically significant *p*‐values.

**TABLE 4:** Association of RAS mutation status and CD147 expression in men patient's subgroup.

Men (*n* = 58)			
Molecular features	CD147 IHC negative (*n* = 23)	CD147 IHC positive (*n* = 35)	*p*
	No of patients (%)	No of patients (%)	
**RAS mutation**			
No	19 (83)	16 (46)	0.005*
Yes	4 (17)	19 (54)	
NI	3	0	

Abbreviation: IHC, immunohistochemistry.

* Statistically significant *p*‐values.

## 4 Discussion

Despite low occurrence rates, LR of rectal adenocarcinoma after surgery appears as a significant health challenge, in particular, because of the associated risk of general dissemination and the difficulties of surgical management of these events [18]. LR often arises from incomplete resection or inadequate neoadjuvant therapy response, while regional and distant recurrences result from micrometastases and systemic spread.

Numerous factors, including surgical, clinical, and pathological tumor attributes, influence the likelihood of recurrence [19]. The positivity of the circumferential resection margins, as well as a clinical and pathological advanced tumor stage and a positive lymph node involvement, represents the main clinical and pathological parameters frequently identified as risk factors for recurrence [20, 21, 22]. Our observations are consistent with previous reports regarding the ypN stage and increased risk of LR [17, 23], with a significantly higher distribution of ypN2 tumors in the LR‐positive group and an associated median number of positive nodes higher than in the LR‐negative group. Interestingly, the ypN0/N1 status distribution was not different between the two groups, suggesting that the number of invaded lymph nodes is a more reliable LR‐predictive marker than the simple positive/negative lymph node classification.

No other parameters (excluded from the initial matching process) were significantly associated with LR, including the adapted PREDICT score or the venous and/or nervous invasion. These negative results may be explained partially by a patient selection bias with regard to initial matching, as well as by the relatively low number of LR‐positive patients included in the study.

Next to clinicopathological features, molecular and genetic tumor characteristics could also represent factors influencing the relapse occurrence. Identifying such factors can aid in highlighting high‐risk patients who may benefit from more intensive postoperative monitoring and additional therapeutic interventions. This work explored for the first time the expression of CD147, a transmembrane protein that plays a significant role in promoting colorectal tumor invasion and metastasis, as a potent biomarker for LR risk evaluation. Thanks to the activation of metalloproteases, CD147 helps tumors overcome natural barriers like the basement membrane and reach lymphatic and blood vessels, allowing them to spread locally and systematically [24]. Previous studies have established CD147 as a prognostic marker in multiple tumor types [9], including CRC [25]. In a study analyzing 328 CRC cases, high CD147 expression was found significantly linked to tumor invasion, metastasis, and TNM stage, as well as associated with a poorer prognosis [26]. However, data available on RC are limited as well as studies focusing on recurrence. Only one study established the association between a high CD147 expression and a higher risk of post‐transplantation recurrence in the context of hepatocellular carcinoma [10]. The present study did not identify any association between CD147 expression and LR rates. Thus, while CD147 expression is a well‐established factor of global poorer prognosis in CRC, it does not seem associated with RC LR. However, the very low LR rates currently observed with RC‐standardized treatment modalities, and the associated small cohorts of patients, leave little room for the identification of new predictive biomarkers. Nevertheless, the identification of biological profiles linked to LR might be achievable by employing broad genomic or proteomic approaches. Many studies have applied these technologies to the prediction of RC response to nCRT, but leaving aside the recurrences aspect, whether local or metastatic [27, 28, 29]. Moreover, as CD147 biological activity is also greatly involved in the metastatic process, it might be interesting to explore its role in RC distant recurrences, which occur more frequently.

Considering the reported influence of oncogene mutations on CRC and RC recurrence, we also examined the mutational status of key CRC‐related oncogenes (*KRAS*, *NRAS*, *BRAF*, and *PIK3CA*) and investigated their correlation with LR. Our results are broadly in line with those reported in the literature regarding mutation frequency [29, 30]. Interestingly, recent publications describe CRC mutational status and sex‐related differences [31, 32]. For instance, women's tumors harbor more *RAS* mutations compared to men's, and our data also confirmed this observation. On the other hand, *RAS* mutations, and especially *KRAS* mutations, are usually associated with advanced disease, distant metastases, and poorer prognosis for CRC [33, 34]. Other studies focusing on postoperative CRC recurrence found *KRAS* mutation status and early recurrence significantly associated [35]. Our data did not demonstrate any impact of *RAS* mutational status on RC LR occurrence. Additionally, we did not report any association between *PIK3CA* mutations and LR occurrence, contrary to what may have been reported by others [36].

Although gender impacts mutation frequencies in CRC, its association with specific mutations also appears to influence patients' prognosis and outcome. As an example, mutations of codon 13, but not codon 12, of *KRAS* were associated with a significant reduction in cancer‐specific survival in women, but not in men [37]. In contrast, a recent study shows that *KRAS‐activating* mutations are associated with poor prognosis for male patients, but not for women [38]. Authors elucidate partially this phenomenon by identifying a *KRAS*‐mediated regulation of Y chromosome‐encoded genes involved in epigenetic homeostasis. Interestingly, we report here a significant correlation between the high expression of CD147 and the presence of *a RAS* mutation (*p* = 0.049), particularly in male patients (*p* < 0.005). The analysis of an independent validation cohort aligned with this observation, showing an association in the male patient subgroup only (*p* = 0.048). In addition to these observations, a key research study highlighted an upregulation of CD147 expression on the surface of pancreatic, lung, and colon tumor cell lines harboring *KRAS* gene mutations compared to *KRAS wild‐type* cells [39]. As other evidence indicates that the MEK–ERK pathway also regulates CD147 expression [40], these findings suggest the possibility of a link between MAP Kinase pathway constitutive activation and CD147 expression upregulation. Interestingly, in the men's subgroup of the validation cohort, we observed a stronger association between CD147 expression and mutational status when incorporating not only *RAS* but also *BRAF* oncogenic mutations in the analysis (*p* = 0.012). Thus, the aggressiveness of CRC driven by constitutive *RAS* activation, and potentially by *BRAF*, may partially be mediated through the upregulation of CD147 expression, particularly in male tumors. However, the validation of this assumption can only be achieved through dedicated experimental analyses, including cellular experiments, which are outside the scope of the current work. Our observations still further reinforce the emerging idea of orienting CRC translational study design and analysis with consideration for gender, leading in the future to potential sex‐related personalized management strategies.

## 5 Conclusion

We report here the first study focusing on the value of CD147 expression, coupled with mutational status, as a potent predictive biomarker of RC post‐surgery LR. No statistical link has been highlighted between CD147 expression or mutational status and recurrence occurrence. However, we confirmed lymph node involvement as a robustly associated factor for RC LR. Finally, we highlighted a significant correlation between *RAS*‐positive mutant status and CD147 expression in men's rectal tumors. Despite some limitations, this work opens up interesting perspectives on the influence of *RAS* mutational status on tumor CD147 functions and more generally on colorectal tumor sex‐linked biology.

## Author Contributions

**Anaëlle Isnard:** formal analysis (equal), investigation (equal), visualization (equal), writing – original draft (equal), writing – review and editing (equal). **Laure‐Amandine Cramier:** investigation (equal), writing – review and editing (equal). **Rémi Vergara:** investigation (equal), writing – review and editing (equal). **Mehdi Boubaddi:** investigation (equal), writing – review and editing (equal). **Donatien Fouche:** investigation (equal), writing – review and editing (equal). **Benjamin Fernandez:** writing – review and editing (equal). **Nathalie Senant:** investigation (equal), writing – review and editing (equal). **Quentin Denost:** conceptualization (equal), methodology (equal), writing – review and editing (equal). **Éric Rullier:** writing – review and editing (equal). **Pierre Dubus:** funding acquisition (equal), writing – review and editing (equal). **Sandrine Dabernat:** writing – review and editing (equal). **Charles Dupin:** methodology (equal), visualization (equal), writing – review and editing (equal). **Véronique Vendrely:** methodology (equal), writing – review and editing (equal). **Anne Rullier:** conceptualization (equal), investigation (equal), methodology (equal), writing – review and editing (equal). **Samuel Amintas:** conceptualization (equal), data curation (equal), formal analysis (equal), funding acquisition (equal), investigation (equal), methodology (equal), resources (equal), supervision (equal), validation (equal), visualization (equal), writing – original draft (equal), writing – review and editing (equal).

## Conflicts of Interest

The authors declare no conflicts of interest.

## Supporting information


**Data S1.** cam471087‐sup‐0001‐DataS1

**Data S2.** cam471087‐sup‐0002‐DataS2

## Data Availability

The data that support the findings of this study are available from the corresponding author upon request.
