# Supplementary material for: Association Between CD147 Expression, RAS Mutational Status, and Local Recurrence in Resected Locally Advanced Rectal Cancer
Source: Cancer Med. 2025 Jul 29;14(15):e71087. doi: 10.1002/cam4.71087 (PMC12305349; doi:10.1002/cam4.71087)
Supplement: Supplementary file 2 — Data S2. cam471087‐sup‐0002‐DataS2 [file CAM4-14-e71087-s001.docx]

**Supplemental Figure 1:** *Flow chart of patients’ selection.*

**
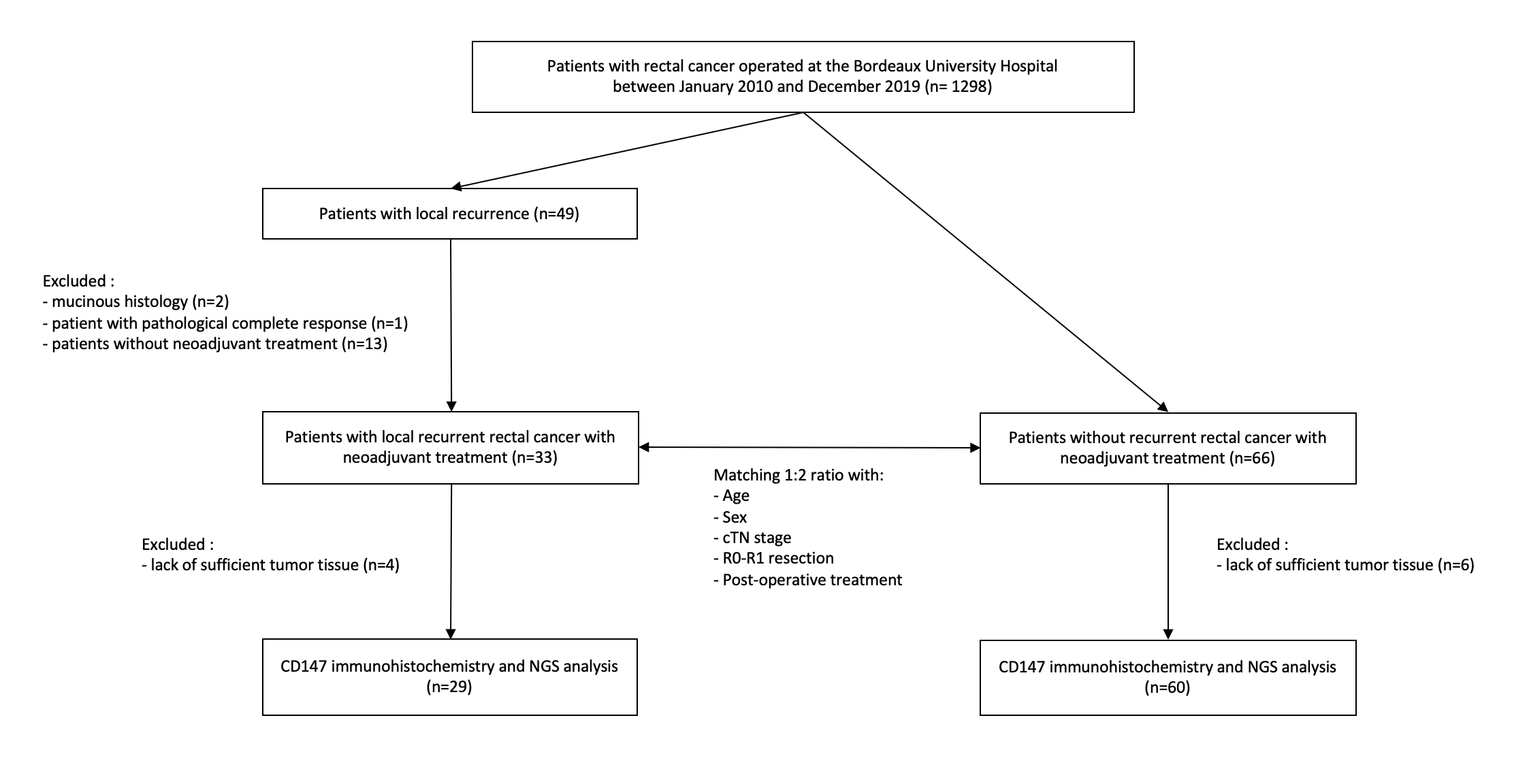
**

**Supplemental Table 1:** *Adapted PREDICT Score.*

| ADAPTED PREDICT SCORE | |
| --- | --- |
| Score item | **Score points** |
| Pathological T3–4 stage | 3 |
| Pathological N1–2 Stage | 2 |
| Tumor < 5 cm from anal verge | 1 |
| Mucinous histology | 2 |
| R1 status | 2 |
| Positive distal resection margins | 1 |
| Lymphovascular invasion | 2 |
| Lack of neoadjuvant therapy | 2 |

*Note: Score ranges from 0 to 15. 0–4 low probability; 5–9 moderate probability; 10–15 high probability of locoregional recurrence.*

**Supplemental Table 2:** *Distribution of CD147 expression staining in reccurent and non-reccurent patients .*

| ALL PATIENTS (n=89) | | | |
| --- | --- | --- | --- |
| **CD147 IHC expression** | **Recurrent (n = 29)**  **No of patients (%)** | **Non-reccurent (n = 60)**  **No of patients (%)** | ***P value*** |
|  | |  |  |
| High | 3 (10.4) | 17 (28.4) | 0.132 |
| Medium | 10 (34.5) | 22 (36.6) |  |
| Low | 11 (37.9) | 17 (28.4) |  |
| Nul | 5 (17.2) | 4 (6.6) |  |

**Supplemental Table 3:** *List of somatic activating mutations identified for KRAS, NRAS, BRAF, and PIK3CA genes in recurrent and non-recurrent patients’ rectal cancer tumors.*

| **Gene** | **Exon** | **Transcript** | **Chr** | **Alt** | **c.** | **p.** | **Number of patients** |
| --- | --- | --- | --- | --- | --- | --- | --- |
| *KRAS* | 2 | NM_033360.4 | chr12 | 25398285 | c.34G>A | p.(Gly12Ser) | 2 |
| *KRAS* | 2 | NM_033360.4 | chr12 | 25398285 | c.34G>T | p.(Gly12Cys) | 1 |
| *KRAS* | 2 | NM_033360.4 | chr12 | 25398284 | c.35G>A | p.(Gly12Asp) | 12 |
| *KRAS* | 2 | NM_033360.4 | chr12 | 25398284 | c.35G>C | p.(Gly12Ala) | 3 |
| *KRAS* | 2 | NM_033360.4 | chr12 | 25398284 | c.35G>T | p.(Gly12Val) | 7 |
| *KRAS* | 2 | NM_033360.4 | chr12 | 25398281 | c.38G>A | p.(Gly13Asp) | 7 |
| *KRAS* | 2 | NM_033360.4 | chr12 | 25398255 | c.64C>A | p.(Gln22Lys) | 1 |
| *KRAS* | 3 | NM_033360.4 | chr12 | 25380282 | c.176C>T | p.(Ala59Val) | 1 |
| *KRAS* | 4 | NM_033360.4 | chr12 | 25378647 | c.351A>T | p.(Lys117Asn) | 1 |
| *KRAS* | 4 | NM_033360.4 | chr12 | 25378643 | c.355G>A | p.(Asp119Asn) | 1 |
| *NRAS* | 2 | NM_002524.5 | chr1 | 115258747 | c.35G>A | p.(Gly12Asp) | 1 |
| *NRAS* | 2 | NM_002524.5 | chr1 | 115258747 | c.35G>T | p.(Gly12Val) | 1 |
| *NRAS* | 2 | NM_002524.5 | chr1 | 115258745 | c.37G>C | p.(Gly13Arg) | 1 |
| *NRAS* | 3 | NM_002524.5 | chr1 | 115256529 | c.182A>G | p.(Gln61Asp) | 1 |
| *BRAF* | 11 | NM_004333.6 | chr7 | 140481403 | c.1405G>C | p.(Gly469Arg) | 1 |
| *PIK3CA* | 10 | NM_006218.4 | chr3 | 178936082 | c.1624G>A | p.(Glu542Lys) | 1 |
| *PIK3CA* | 10 | NM_006218.4 | chr3 | 178936091 | c.1633G>A | p.(Glu545Lys) | 5 |
| *PIK3CA* | 10 | NM_006218.4 | chr3 | 178936095 | c.1637A>G | p.(Gln546Arg) | 1 |
| *PIK3CA* | 10 | NM_006218.4 | chr3 | 178936103 | c.1645G>A | p.(Asp549Asn) | 1 |
| *PIK3CA* | 21 | NM_006218.4 | chr3 | 178952049 | c.3104C>T | p.(Ala1035Val) | 1 |
| *PIK3CA* | 21 | NM_006218.4 | chr3 | 178952084 | c.3139C>T | p.(His1047Tyr) | 3 |
| *PIK3CA* | 21 | NM_006218.4 | chr3 | 178952085 | c.3140A>G | p.(His1047Arg) | 2 |

Chr: chromosome; Alt: Altered genomic base position

**Supplemental Table 4:** *Clinicopathological characteristics, CD147 IHC expression, and RAS/BRAF mutation status of the validation cohort.*

| **ALL PATIENTS = 86** | |
| --- | --- |
| **Clinicopathological features** | **Number of patients (%)** |
| **Age, years** |  |
| <65 | 43 (50) |
| ≥65 | 43 (50) |
| **Sex** |  |
| Male | 55 (64) |
| Female | 31 (36) |
| **Distant tumor from anal verge (cm)** |  |
| <5 | 33 (38) |
| ≥5 | 52 (62) |
| **cT stage** |  |
| cT1  cT2 | 5 (6)  10 (12) |
| cT3 | 52 (60) |
| cT4 | 17 (20) |
| cTx | 2 (2) |
| **ypT stage** |  |
| ypT1 | 10 (12) |
| ypT2 | 11 (13) |
| ypT3 | 55 (63) |
| ypT4 | 10 (12) |
| **ypN stage** |  |
| ypN0 | 32 (37) |
| ypN+  ypNx | 53 (62)  1 (1) |
|  |  |
| **Neoadjuvant treament** |  |
| **No** | 27 (31) |
| **Yes** | 59 (69) |
| **Anatomopathological tumor size (cm)** | |
| <3 | 30 (35) |
| ≥3 | 56 (65) |
| **Differenciation** | |
| Well-moderate | 82 (95) |
| Poor | 4 (5) |
| **Resection** |  |
| R0 | 68 (79) |
| R1 | 18 (21) |
| **Vascular and/or nervous invasion** | |
| **No** | 33 (38) |
| **Yes** | 57 (62) |
| Vascular invasion | 26 (46) |
| Nervous invasion | 8 (14) |
| Both | 19 (33) |
| **Distal resection margins status** |  |
| Negative | 82 (95) |
| Positive | 4 (5) |
| ***RAS* or *BRAF* mutation** |  |
| **No** | 33 (38) |
| **Yes** | 53 (62) |
| *KRAS* | 43 (50) |
| *NRAS* | 5 (6) |
| *BRAF* | 5 (6) |
| ***CD147 IHC expression*** |  |
| *Positive* | 62 (72) |
| *Negative* | 24 (28) |
|  |  |

*RAS : KRAS or NRAS, IHC : Immunohistochemistry*

**Supplemental Table 5:** *Patients’ local recurrence characteristics*

| RECCURENCE POSITIVE PATIENTS (n=29) | | |
| --- | --- | --- |
| Recurrence localization | **No of patients (%)** | **Local recurrence treatment type** |
| Local Regrowth | 29 (100) |  |
| *Pelvic** | 24 (83) | 14 APR + CT  10 PCT |
| *Intraluminal*** | 5 (17) | 2 LE  1 APR  1 TME  1 PCT |
| Associated metastatic localization | 16 (55) |  |

**Pelvis or mesorectum, **anastomotic or regrowth after local excision,* LE: Local Excision, APR: Abdomino Perineal Resection, TME: Total Mesorectal Resection, PCT: Palliative chemotherapy, CT: Chemotherapy.

**Supplemental Table 6:** *Patients’ associated metastatic recurrence characteristics*

| RECCURENCE POSITIVE PATIENTS (n=29) | | |
| --- | --- | --- |
| Recurrence localization | **No of patients (%)** | **Metastatic recurrence treatment type** |
| Lungs + liver | 2 (7) | 1 CT  1 PCT |
| Lungs | 5 (17) | 2 PCT  1 CT  1 RF  1 surgery |
| Liver | 6 (21) | 2 PCT  2 Surgery  1 CT  1 RF |
| Peritoneum | 2 (7) | 2 PCT |
| Lumbo - Aortic Lymph node | 1 (3) | 1 PCT |

PCT: Palliative chemotherapy, CT: Chemotherapy, RF: Radiofrequency

**Supplemental Table 7:** *Association of recurrence with patients’ quantitative clinicopathological features.*

| ALL PATIENTS (n=89) | | | |
| --- | --- | --- | --- |
| **Clinicopathological features** | **Recurrent (n= 29)**  **Mean ± sd** | **Nonrecurrent (n=60) Mean ± sd** | ***P value*** |
| Median clinical tumor size (cm) | 4.9 ± 1.7 | 5.0 ± 1.9 | 0.88 |
| Median anatomopathological tumor size (cm) | 3.2 ± 1.7 | 3.1 ± 2.0 | 0.74 |
| Median lymph nodes examined | 17.6 ± 7.3 | 19.6 ± 8.5 | 0.3 |
| Median lymph nodes involved | 2.4 ± 3.5 | 0.9 ± 1.5 | **0.008*** |
| Anal margin height (cm) | 4.98 ± 2.48 | 4.84 ± 2.46 | 0.81 |

sd: standard deviation.

**Supplemental Table 8:** *Association of sex and tumors mutational status.*

| ALL PATIENTS (n=89) | | | |
| --- | --- | --- | --- |
| **Molecular features** | **Female (n= 25)  No of patients (%)** | **Male (n=58)  No of patients (%)** | ***P value*** |
| **Mutation** | |  |  |
| No | 8 (32) | 27 (47) | 0.218 |
| Yes | 17 (68) | 31 (53) |  |
| NI | 3 | 3 |  |
| **Number of mutations** | |  |  |
| 0 | 8 (32) | 27 (47) | 0.131 |
| 1 | 11 (44) | 26 (45) |  |
| ≥2 | 6 (24) | 5 (8) |  |
| NI | 3 | 3 |  |
| ***RAS* mutation** | |  |  |
| No | 8 (32) | 35 (60) | **0.018*** |
| Yes | 17 (68) | 23 (40) |  |
| NI | 3 | 3 |  |
| ***PIK3CA* mutation** | |  |  |
| No | 19 (76) | 50 (86) | 0.255 |
| Yes | 6 (24) | 8 (14) |  |
| NI | 3 | 3 |  |

**Supplemental Table 9:** *Association of recurrence and tumors mutational status.*

| ALL PATIENTS (n=89) | | | |
| --- | --- | --- | --- |
| **Molecular features** | **Recurrent (n= 29)  No of patients (%)** | **Nonrecurrent (n=60)  No of patients (%)** | ***P value*** |
| **Presence of mutation** |  |  |  |
| No | 12 (46) | 23 (40) | 0.62 |
| Yes | 14 (54) | 34 (60) |  |
| NI | 3 | 3 |  |
| **Number of mutations** |  |  |  |
| 0 | 12 (46) | 23 (40) | 0.265 |
| 1 | 13 (50) | 24 (42) |  |
| ≥2 | 1 (4) | 10 (18) |  |
| NI | 3 | 3 |  |
| ***RAS* mutation** |  |  |  |
| No | 17 (65) | 26 (46) | 0.095 |
| Yes | 9 (35) | 31 (54) |  |
| NI | 3 | 3 |  |
| ***PIK3CA* mutation** |  |  |  |
| No | 23 (88) | 46 (81) | 0.381 |
| Yes | 3 (12) | 11 (19) |  |
| NI | 3 | 3 |  |

**Supplemental Table 10:** *Association of RAS mutation status and CD147 expression in women patient’s subgroup.*

| WOMEN (n=31) | | | |
| --- | --- | --- | --- |
| **Molecular features** | **CD147 IHC negative (n = 14)**  **No of patients (%)** | **CD147 IHC positive (n = 17)**  **No of patients (%)** | ***P value*** |
| ***RAS* mutation** |  |  |  |
| No | 3 (25) | 5 (30) | 0.653 |
| Yes | 8 (75) | 9 (70) |  |
| NI | 3 | 0 |  |

IHC : Immunohistochemistry

**Supplemental Table 11:** *Association of RAS mutation status and CD147 expression in women patient’s subgroup of the validation cohort****.***

| WOMEN (n=31) | | | |
| --- | --- | --- | --- |
| **Molecular features** | **CD147 IHC negative (n = 10)**  **No of patients (%)** | **CD147 IHC positive (n = 21)**  **No of patients (%)** | ***P value*** |
| ***RAS* mutation** |  |  |  |
| No | 4 (40) | 11 (52) | 0.519 |
| Yes | 6 (60) | 10 (48) |  |
|  |  |  |  |

IHC : Immunohistochemistry

**Supplemental Table 12:** *Association of RAS mutation status and CD147 expression in men patient’s subgroup of the validation cohort****.***

| MEN (n=55) | | | |
| --- | --- | --- | --- |
| **Molecular features** | **CD147 IHC negative (n = 14)**  **No of patients (%)** | **CD147 IHC positive (n = 41)**  **No of patients (%)** | ***P value*** |
| ***RAS* mutation** |  |  |  |
| No | 9 (64) | 14 (34) | **0.048*** |
| Yes | 5 (36) | 27 (66) |  |

IHC : Immunohistochemistry

**Supplemental Table 13:** *Association of RAS and BRAF mutation status and CD147 expression in men patient’s subgroup of the validation cohort****.***

| MEN (n=55) | | | |
| --- | --- | --- | --- |
| **Molecular features** | **CD147 IHC negative (n = 14)**  **No of patients (%)** | **CD147 IHC positive (n = 41)**  **No of patients (%)** | ***P value*** |
| ***RAS* or BRAF mutation** |  |  |  |
| No | 9 (64) | 11 (33) | **0.012*** |
| Yes | 5 (36) | 30 (67) |  |

IHC : Immunohistochemistry
